# Deep Divergence and Genomic Diversification of Gut Symbionts of Neotropical Stingless Bees

**DOI:** 10.1128/mbio.03538-22

**Published:** 2023-03-20

**Authors:** Garance Sarton-Lohéac, Carlos Gustavo Nunes da Silva, Florent Mazel, Gilles Baud, Vincent de Bakker, Sudip Das, Yassine El Chazli, Kirsten Ellegaard, Marc Garcia-Garcera, Natasha Glover, Joanito Liberti, Lorena Nacif Marçal, Aiswarya Prasad, Vincent Somerville, SAGE class 2019-2020 and 2020-2021, Germán Bonilla-Rosso, Philipp Engel

**Affiliations:** a Department of Fundamental Microbiology, University of Lausanne, Lausanne, Switzerland; b Department of Ecology and Evolution, University of Lausanne, Lausanne, Switzerland; c Swiss Institute of Bioinformatics, Lausanne, Switzerland; d Société des produits Nestlé, Lausanne, Switzerland; e Department of Morphology, Instituto de Ciências Biológicas, Federal University of Amazonas, Manaus, Brazil; University of Connecticut

**Keywords:** bacteria, diversification, genome, gut microbiome, insects, phylogeny, stingless bee, symbiosis

## Abstract

Social bees harbor conserved gut microbiotas that may have been acquired in a common ancestor of social bees and subsequently codiversified with their hosts. However, most of this knowledge is based on studies on the gut microbiotas of honey bees and bumblebees. Much less is known about the gut microbiotas of the third and most diverse group of social bees, the stingless bees. Specifically, the absence of genomic data from their microbiotas presents an important knowledge gap in understanding the evolution and functional diversity of the social bee microbiota. Here, we combined community profiling with culturing and genome sequencing of gut bacteria from six neotropical stingless bee species from Brazil. Phylogenomic analyses show that most stingless bee gut isolates form deep-branching sister clades of core members of the honey bee and bumblebee gut microbiota with conserved functional capabilities, confirming the common ancestry and ecology of their microbiota. However, our bacterial phylogenies were not congruent with those of the host, indicating that the evolution of the social bee gut microbiota was not driven by strict codiversification but included host switches and independent symbiont gain and losses. Finally, as reported for the honey bee and bumblebee microbiotas, we found substantial genomic divergence among strains of stingless bee gut bacteria, suggesting adaptation to different host species and glycan niches. Our study offers first insights into the genomic diversity of the stingless bee microbiota and highlights the need for broader samplings to understand the evolution of the social bee gut microbiota.

## INTRODUCTION

The eusocial corbiculate bees (referred to here as social bees) comprise more than 700 species distributed in three distinct tribes: honey bees (Apini), bumblebees (Bombini), and stingless bees (Meliponini). Stingless bees and bumblebees form a monophyletic clade, which is sister to the honey bees, with whom they shared a common ancestor 80 to 100 million years ago (1–3). Like mammals, social bees harbor dense and specialized bacterial communities in their gut that affect bee health and behavior (4–12). The composition of the gut microbiota of social bees is relatively simple, typically consisting of <10 bacterial phylotypes, i.e., sequence clusters sharing >97% identity in the 16S rRNA gene (13–20). Five of these phylotypes (*Snodgrassella*, *Gilliamella*, *Bombilactobacillus* Firm-4, *Lactobacillus* Firm-5, and *Bifidobacterium*) have been referred to as the core gut microbiota of the social bees (20), because they are prevalent and abundant across honey bees, bumblebees, and stingless bees. Most members of the bee gut microbiota are culturable, and gnotobiotic bees can be generated for several species (5, 21–23). Together, these distinctive characteristics make the social bee microbiota a versatile model system for studying the evolution and ecology of host-associated microbial communities. Moreover, social bees are important pollinators that suffer from severe population declines (24, 25) which makes studies of their microbiota relevant in their own right.

Most of what is currently known about the gut microbiota of social bees stems from studies on honey bees and bumblebees. Genomic and experimental approaches have revealed that their gut bacteria are usually saccharolytic fermenters that utilize plant glycans derived from the pollen and nectar/honey diet of the host (22, 23, 26–32). Further, it has been shown that the core members of the honey bee and bumblebee gut microbiotas have substantially diversified (27–30, 33–36). They consist of divergent sublineages (or species) and exhibit extensive strain-level diversity and gene content variation. Most sublineages are host specific (23, 27, 33, 36), and their phylogenetic relationships are to some extent congruent with the phylogeny of the host (16, 33). Therefore, it has been suggested that the core members of the microbiota were acquired in a common ancestor of the social bees (20) and possibly codiversified with the host (16, 33). In addition, studies in the Western honey bee (Apis mellifera) have shown that the diversification of the bee gut microbiota was also driven by adaptation to different spatial and metabolic niches within the gut (27, 30, 33–35). For example, strains of closely related sublineages of *Lactobacillus* Firm-5 and *Bifidobacterium* can coexist in individual bees. They carry distinct gene sets for the breakdown and utilization of pollen-derived carbohydrates, which allows them to partition the available dietary glycan niches in the gut (27, 30, 34).

In contrast to the microbiotas of honey bees and bumblebees, much less is known about the gut microbiota of the third group of social bees, the stingless bees (Meliponini). Previous studies have focused on determining the taxonomic composition of the gut microbiota of these bees using 16S rRNA gene sequencing (15, 17, 20, 37–39). However, only a few bacteria have been cultured from stingless bees (40–42), and except for two strains of *Bombilactobacillus* Firm-4 recently isolated from bees from Australia (43), no genomic data are currently available for core members of the gut microbiota of stingless bees.

With >500 described species, stingless bees present the largest and most diverse group of the social bees (44, 45). They are naturally distributed throughout the tropical and subtropical regions of Africa, Asia, Australia, and the Americas and exhibit great variation in morphology, diet, foraging range, social structure, and nesting habits (44, 45). As host phylogeny and ecology are both key determinants of gut microbiota composition (46–51), we hypothesize that genomic studies on bacterial isolates will help us to understand the functional diversity of gut bacteria of stingless bees and provide novel insights into the evolution of these bacteria across social bees, specifically in respect of the possible codiversification with the host.

To address these questions, we looked at the gut microbiotas of six neotropical species of stingless bees from Brazil: Frieseomelitta varia (Fv), Scaptotrigona polysticta (Sp), Melipona fuliginosa (Mf), Melipona interrupta (Mi), Melipona seminigra (Ms), and Melipona lateralis (Ml). We determined the composition of the gut microbiota of these bees using 16S rRNA gene sequencing, established a comprehensive culture collection of bacterial isolates, and conducted genome sequencing and comparative genomics to determine the phylogenetic placement, genomic diversity, and functional capabilities of these bacteria relative to those previously isolated from honey bees and bumblebees.

## RESULTS

### Six neotropical stingless bee species from Brazil harbor distinct gut microbiotas dominated by nine bacterial families.

**FIG 1:**
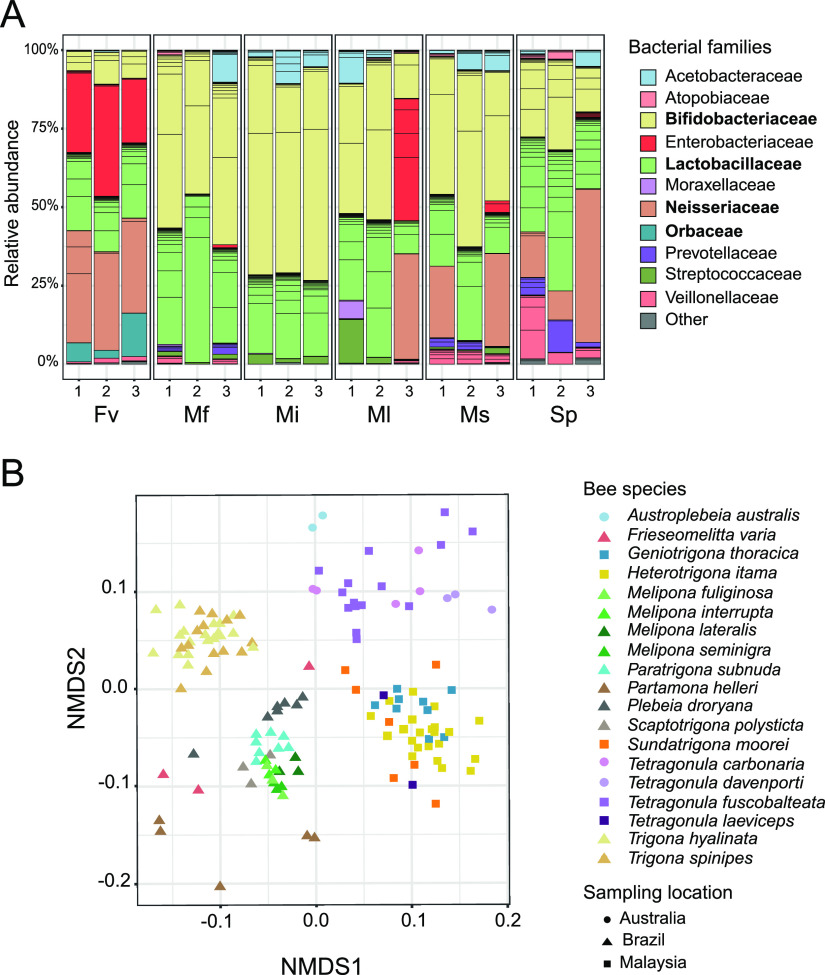
Community analysis of the gut microbiotas of stingless bees. (A) 16S rRNA gene-based community profiles of the gut microbiotas of three colonies of six stingless bee species collected in Brazil. Fv, *Frieseomelitta varia*; Ms, *Melipona seminigra*; Ml, *Melipona lateralis*; Mf, *Melipona fuliginosa*; Mi, *Melipona interrupta*; Sp, *Scaptotrigona polysticta*. Relative abundance of ASVs is shown. ASVs are ordered and colored at the family level (see the key); families of core members are in bold. ASVs with <1% relative abundances are summed up as “Other” and shown in gray. (B) NMDS based on ASV relative abundance (Bray-Curtis dissimilarity) across 136 16S rRNA gene amplicon sequence samples, including data from a previous study (20) and our study.

We sampled three colonies of six stingless bee species (Fv, Sp, Mf, Mi, Ms, and Ml) from a meliponary located in the Amazonian rainforest near Manaus (see Table S1 in the supplemental material). For each colony, we pooled the guts of 15 to 60 worker bees (depending on the size of the bee species; see Materials and Methods) before DNA isolation. The V4 region of the 16S rRNA gene was amplified and sequenced with the Illumina MiSeq 2×250-bp platform, resulting in a median depth of 64,713 (52,479 to 95,774) reads per sample. In total, we identified 277 amplicon sequence variants (ASVs; 29 to 63 ASVs per sample), which belonged to 36 different bacterial families (Table S2). Despite this diversity, only nine families dominated the samples, together representing 97% of all quality-filtered reads (93 to 99% of the reads per sample): *Acetobacteraceae*, *Bifidobacteriaceae*, *Enterobacteriaceae*, *Lactobacillaceae*, *Neisseriaceae*, *Orbaceae*, *Prevotellaceae*, *Streptococcaceae*, and *Veillonellaceae* (Fig. 1A).

10.1128/mbio.03538-22.7 TABLE S1 Gut homogenate metadata of the 18 stingless bee hives. For each bee species, individuals were collected from three hives in a rural meliponary. Download Table S1, XLSX file, 0.01 MB. Copyright © 2023 Sarton-Lohéac et al. 2023 Sarton-Lohéac et al. https://creativecommons.org/licenses/by/4.0/ This content is distributed under the terms of the Creative Commons Attribution 4.0 International license.

10.1128/mbio.03538-22.8 TABLE S2 ASV table of our 18 samples. For each ASV, the assigned taxonomy and the number of reads in each sample are displayed. In the sheet RelAbundance, read counts were transformed to relative abundance for each sample. In the sheet Absence_Presence, 1 indicates that at least one read matched the ASV, and 0 indicates that the ASV was not found in the sample. Finally, the Family_relAbundaces tab provides a summary of the ASV abundance from each taxonomic family per sample. Download Table S2, XLSX file, 0.1 MB. Copyright © 2023 Sarton-Lohéac et al. 2023 Sarton-Lohéac et al. https://creativecommons.org/licenses/by/4.0/ This content is distributed under the terms of the Creative Commons Attribution 4.0 International license.

While *Lactobacillaceae* and *Bifidobacteriaceae* were abundant across all samples, there were clear differences in the distribution of some of the other bacterial families (Fig. 1A). *Neisseriaceae* were abundant in the samples of Sp and Fv but were detected in only three of 12 samples from the genus *Melipona* (Fig. 1A; Table S2). In contrast, *Acetobacteraceae* and *Streptococcaceae* were present in most *Melipona* samples but rare across samples from Fv and Sp. *Orbaceae* and *Enterobacteriaceae* were mostly detected in the three Fv samples. Intriguingly, a single *Enterobacteriaceae* ASV constituted the most abundant community member in this bee species (21 to 36% of the reads per sample). According to these compositional differences, nonmetric multidimensional scaling (NMDS) separated the samples into three distinct clusters: two clusters comprised all samples from Fv and Sp, and the third cluster comprised all samples from the four *Melipona* species (Mf, Mi, Ms, and Ml) (Fig. S1A).

10.1128/mbio.03538-22.1 FIG S1 (A) NMDS based on ASV relative abundance in the 18 samples collected from six different stingless bee species in Brazil. The samples clustered by bee genus (PERMANOVA: genus pseudo-*F* = 11.05, *P* < 0.001). (B) Community profiles of the gut microbiota of 136 samples from 19 different stingless bee species collected in Brazil, Malaysia, and Australia. Relative abundance of ASVs is shown. ASVs are ordered and colored at the genus level according to the legend. ASVs with <1% relative abundances are summed up as “others” and shown in grey. This plot includes the data sets for stingless bees from reference 20 and our study. The samples are ordered by bee species: Aa, Austroplebeia australis; Fv, Frieseomelitta varia; Gt, Geniotrigona thoracica; Hi, Heterotrigona itama; Mf, *Melipona fuliginosa*; Mi, *Melipona interrupta*; Ml, *Melipona lateralis*; Ms, *Melipona seminigra*; Ps, Paratrigona subnuda; Ph, Partamona helleri; Pd, Plebeia droryana; Sp, *Scaptotrigona polysticta*; Tc, Tetragonula carbonaria; Td, Tetragonula davenporti; Tf, Tetragonula fuscobalteata; Tl, Tetragonula laeviceps; Th, Trigona hyalinata; Ts, Trigona spinipes. (C) Number of shared ASVs. For each family, we reported how many ASVs were observed (indicated by the dot size) in how many bee species. Families with an average abundance of <1% are grouped as “Other.” The sum of species-specific or shared (by two or more bee species) ASVs is reported on the right side. Core member families are in bold. (D) Relative abundances of the shared ASVs across the 19 stingless bee species (samples from the same species were pooled). Download FIG S1, EPS file, 14.8 MB. Copyright © 2023 Sarton-Lohéac et al. 2023 Sarton-Lohéac et al. https://creativecommons.org/licenses/by/4.0/ This content is distributed under the terms of the Creative Commons Attribution 4.0 International license.

### Related stingless bee species have overlapping community profiles.

We compared our results to a previously published amplicon sequencing data set from stingless bees (20) to assess the similarity of the communities to those of other stingless bee species. After discarding samples with <5,000 reads to control for variation in sequencing depths, our data set comprised 135 samples from 19 different host species and three different countries. We detected 688 ASVs in total with a median of 18 ASVs per sample (3 to 63 ASVs) (Table S3), spanning 53 bacterial families. Overall, the taxonomic patterns were similar across the analyzed bee species. Apart from one sample from Tetragonula fuscobalteata, for which 99% of the reads belonged to a single *Weeksellaceae* ASV, the nine families dominating in the six bee species from our study were also abundant in the microbiotas of the samples from the previous study and represented 34% to 100% of the total number of reads (Fig. S1B; Table S3).

10.1128/mbio.03538-22.9 TABLE S3 ASV table. For each ASV, the assigned taxonomy and the number of reads in each sample are displayed. This analysis groups our 18 samples and samples from reference 20. In the sheet RelAbundance, read counts were transformed to relative abundance for each sample. In the sheet Absence_Presence, 1 indicates that at least one read matched the ASV, and 0 indicates that the ASV was not found in the sample. Finally, the Family_relAbundaces tab provides a summary of the ASV abundance from each taxonomic family per sample. Download Table S3, XLSX file, 1.0 MB. Copyright © 2023 Sarton-Lohéac et al. 2023 Sarton-Lohéac et al. https://creativecommons.org/licenses/by/4.0/ This content is distributed under the terms of the Creative Commons Attribution 4.0 International license.

NMDS based on ASV relative abundances separated the samples by location (i.e., samples from Brazil were different from those from Australia and Malaysia) (permutational multivariate analysis of variance [PERMANOVA]; location pseudo-*F* = 20.17, *P* = 0.001) and by bee genus (PERMANOVA; genus pseudo-*F* = 10.49, *P* = 0.001), although taxonomy and geography are not independent (Fig. 1B). In contrast, there was only weak clustering at the species level (species pseudo-*F* = 1.96, *P* = 0.03). Notably, only 30% (206 of 688) of all ASVs were shared across host species (i.e., 70% of all ASVs are found in only one species) and most of them (83.4%) only between 2 and 5 species (Fig. S1C). However, the shared ASVs belonged to the nine predominant bacterial families and represented a large fraction of the total number of reads per sample (53.3 to 99.7%; except that for the samples from Fv and Partamona helleri, the fraction was 10.6 to 15% of the reads) (Fig. S1D). In particular, bees sampled in the same country or belonging to the same bee genus shared the same ASVs, explaining the clustering of these samples in the NMDS analysis. Together, these results show that despite the large variability observed, the gut microbiotas of most stingless bee species are dominated by a few bacterial families and that bee species of the same genus, or with overlapping geographic distribution, have similar community profiles at the 16S rRNA gene level.

### Establishment of a strain collection of gut bacteria isolated from stingless bees.

**FIG 2:**
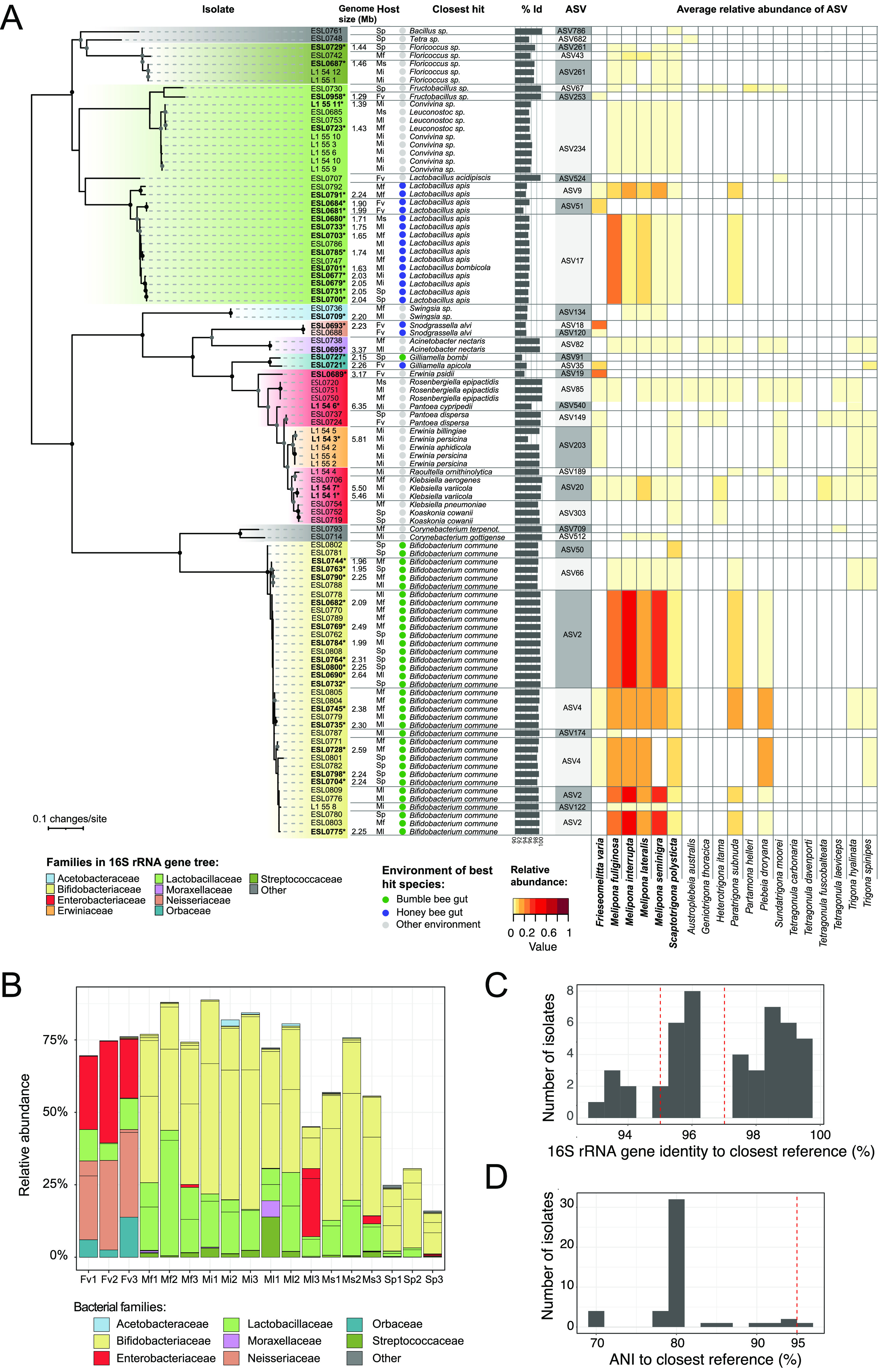
Bacterial strains isolated from the gut of six stingless bee species from Brazil. (A) Maximum-likelihood tree inferred from nearly complete sequences of the 16S rRNA gene of each isolate. Strain names of isolates for which we sequenced the genome are in bold. The genome size of each sequenced strain is noted next to it. The isolate host is indicated as follows: Fv, *Frieseomelitta varia*; Ms, *Melipona seminigra*; Ml, *Melipona lateralis*; Mf, *Melipona fuliginosa*; Mi, *Melipona interrupta*; Sp, *Scaptotrigona polysticta*. “Closest hit” indicates best BLASTN hit of the 16S rRNA gene sequence against the 16S rRNA (NCBI: *Bacteria* and *Archaea* type strains) database. Colored circles indicate if the strain of the best hit was isolated from the gut of a bumblebee or a honey bee or elsewhere. Bar plots indicate percent identity of the best BLASTN hit. The matching ASV and its average relative abundance across the 19 analyzed stingless bee species are indicated. The names of the stingless bee species from our study are in bold. Note that many isolates matched the same ASV. (B) Relative abundances of matching ASVs in each of the 18 samples of the six stingless bee species sampled in our study, i.e., representativeness of the isolates in the amplicon data. (C) Distribution of the isolated strains based on best 16S rRNA gene identity to the closest reference species. These are the values shown in panel A. The dashed lines indicate 95% and 97% identity corresponding to the genus and species thresholds, respectively. (D) Distribution of the isolated strains based on pairwise ANI with the closest reference genome publicly available. The dashed line indicates the species threshold at 95% ANI.

To enable genomic and experimental analyses of stingless bee gut bacteria, we established a culture collection of bacteria isolated from Fv, Sp, Mf, Mi, Ms, and Ml. We plated homogenized gut samples from the six bee species on eight different semisolid media and under three different atmospheres (microaerobic and anaerobic). This resulted in the cultivation of 98 distinct bacterial isolates (i.e., having different 16S rRNA genotypes or isolated from a different bee species or colony) from 11 bacterial families (Fig. 2A; Table S4). Most bacteria grew under both microaerobic and anaerobic conditions on generic growth media and formed colonies after 2 to 4 days of growth. The 16S rRNA genotypes of the isolated strains matched 32 ASVs, accounting for 16 to 87% of the overall community of the six stingless bee species and including many shared ASVs (Fig. 2A and B). BLASTN searches of the 16S rRNA gene sequences revealed that many of the isolates (55/98) were related to bacterial strains obtained from the gut of honey bees and bumblebees (such as Lactobacillus apis, Bifidobacterium commune, *Gilliamella* sp., or Snodgrassella alvi) suggesting that they represent stingless bee isolates of core members of the social bee microbiota. Other isolates had best BLASTN hits to bacteria from other environments, such as a *Floricoccus* sp. isolated from flowers, various *Enterobacteriaceae* (e.g., *Pantoea* sp., Klebsiella sp., and Rosenbergiella epipactidis) isolated from humans and water, or *Fructobacillus* isolated from flowers and fruits. The percent identity of many of the BLAST hits was relatively low (<98%), suggesting that the isolated strains potentially correspond to new bacterial species (Fig. 2A and C; Table S4).

10.1128/mbio.03538-22.10 TABLE S4 Stingless bee strain collection. For each isolate, the bacterial species and bee host are indicated. We subjected the 16S rRNA gene sequences to a BLAST search against the NCBI nr database and extracted for each isolate the reference type strain ID and the percent identity between 16S rRNA gene sequences of the type strain and our isolate. The Sequenced_Isolates tab indicates which strains were sequenced. Download Table S4, XLSX file, 0.03 MB. Copyright © 2023 Sarton-Lohéac et al. 2023 Sarton-Lohéac et al. https://creativecommons.org/licenses/by/4.0/ This content is distributed under the terms of the Creative Commons Attribution 4.0 International license.

### Stingless bee isolates form deep-branching phylogenetic lineages related to bacteria isolated from honey bees and bumblebees.

To assess the phylogenetic placement of the isolated stingless bee gut bacteria relative to gut bacteria from honey bees and bumblebees, we selected 46 strains from 10 different bacterial families for genome sequencing (Fig. 2A; Table S4). Using a combination of Illumina and Oxford Nanopore sequencing, we obtained 23 complete and 23 draft genomes (2 to 66 contigs). The genome size of the cultured isolates ranged from 1.2 to 6.3 Mb. *Fructobacillus* ESL0730 (1.2 Mb) and the two *Streptococcaceae* strains ESL0687 and ESL0729 (1.4 Mb) harbored the smallest and *Leuconostocaceae* ESL0723 the largest (6.3 Mb) genomes of the sequenced strains (Table S4). Genome comparisons with other bacteria, including strains isolated from honey bees and bumblebees, showed that most stingless bee gut bacteria had 80% average nucleotide identity (ANI) with previously sequenced strains indicating that we isolated strains of novel bacterial species or genera (Fig. 2D).

**FIG 3:**
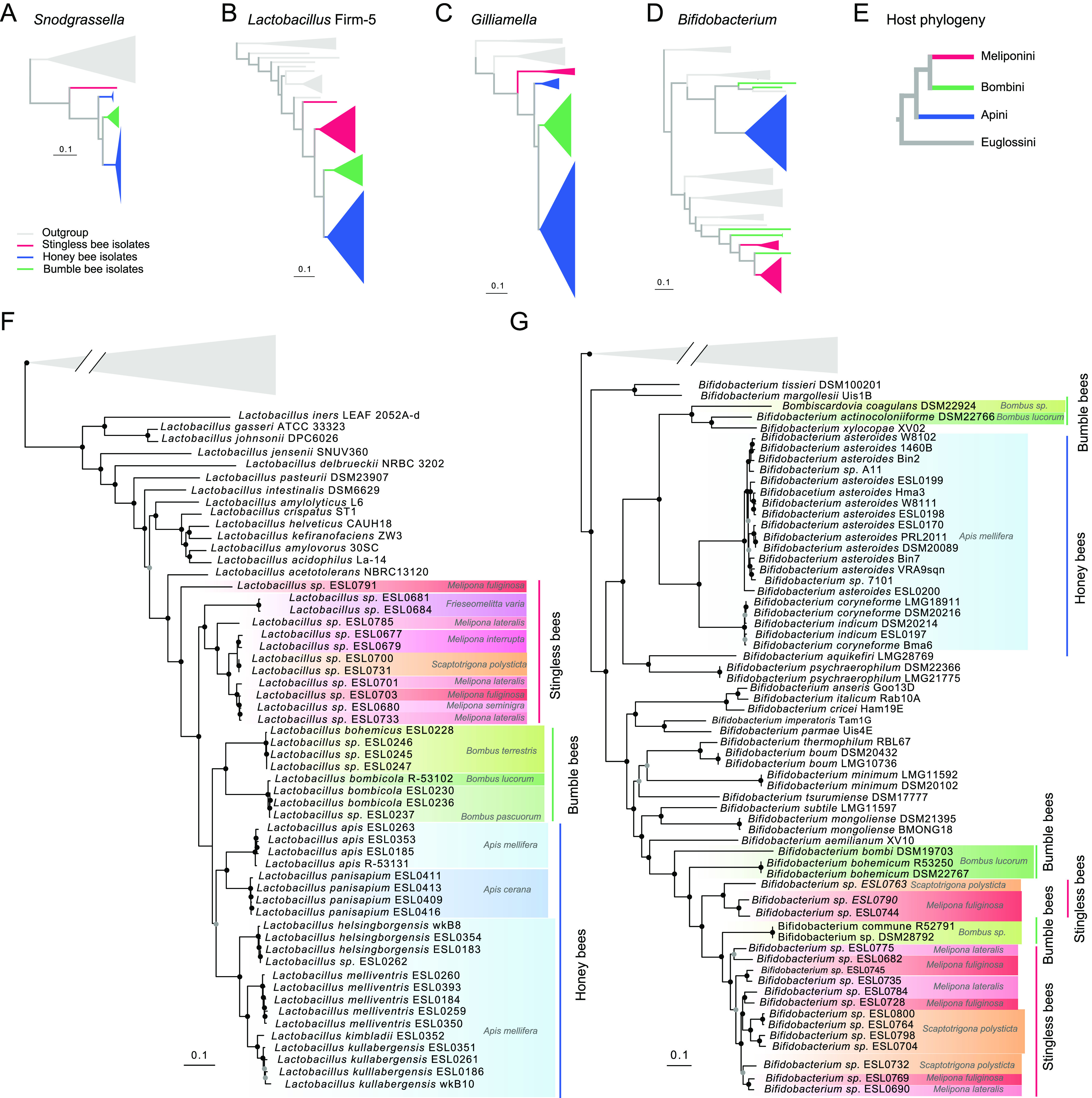
Isolates of stingless bee gut bacteria present novel species and belong to deep-branching phylogenetic lineages. (A to D) Simplified genome-wide maximum-likelihood phylogenies of *Snodgrassella*, *Lactobacillus* Firm-5, *Gilliamella*, and *Bifidobacterium* based on single-copy gene orthologs. All branches shown are supported by >95/100 bootstrap replicates. Most of the branches have been collapsed. (E) Dendrogram depicting the topology of the social bee phylogeny (adapted from references 52 and 53). (F) Detailed genome-wide phylogeny of the genus *Lactobacillus* with bacteria belonging to the social bee-specific phylotype *Lactobacillus* Firm-5 highlighted in different colors according to the host species/group. The maximum-likelihood tree was computed on the concatenated amino acid sequences of 355 single-copy core genes using the substitution model LG+F+I+G4. (G) Detailed genome-wide phylogeny of the genus *Bifidobacterium* with bacteria belonging to social bee-specific clades highlighted in different colors according to the host species/group. The maximum-likelihood tree was computed on the concatenated amino acid sequences of 151 single-copy core genes using the substitution model LG+F+I+G4.

Accordingly, genome-wide phylogenies based on single-copy orthologs showed that most isolates formed deep-branching, stingless bee-specific lineages, exclusive of any previously sequenced strain. However, consistent with the results of the 16S rRNA gene analysis, several of these lineages were related to major phylotypes of the honey bee and bumblebee gut microbiota (Fig. 3A to D; Fig. S2 to 6) such as *Snodgrassella*, *Gilliamella*, *Lactobacillus* Firm-5, *Bifidobacterium*, and *Bombella*. In the case of *Snodgrassella*, *Gilliamella*, and *Lactobacillus* Firm-5, the stingless bee-specific lineages formed a monophyletic clade with lineages of honey bee and bumblebee isolates (Fig. 3A to C and F). Notably, in all three cases, the bacteria from stingless bees presented the earliest-branching lineages, i.e., the honey bee and bumblebee gut bacteria diverged after the split from the stingless bee gut bacteria. While these results suggest that these bacteria are derived from a common ancestor that was already adapted to social bees, the bacterial phylogenies were incongruent with current phylogenies of the host, which show that the honey bees (Apini) diverged before the split of stingless bees (Meliponini) and bumblebees (Bombini) (Fig. 3E). A different pattern was observed for *Bifidobacterium*. In this case, strains isolated from stingless bees, honey bees, and bumblebees were not monophyletic. In fact, the stingless bee isolates belonged to a different clade than the honey bee isolates, while the strains isolated from bumblebees belonged to either of them (Fig. 3D and G). Similarly, the two *Acetobacteraceae* strains (ESL0695 and ESL0709) were not monophyletic with the honey bee isolates of the genus *Bombella*, although they belonged to the same Hymenoptera-associated clade within this family (Fig. S3). This suggests that in both cases, *Bifidobacterium* and *Acetobacteraceae*, bacteria of distinct lineages have independently adapted to the gut environment of social bees.

10.1128/mbio.03538-22.2 FIG S2 (A) Core genome phylogeny of the *Neisseriaceae* including the phylotype *Snodgrassella*. The maximum-likelihood tree was computed with the amino acid sequences of 428 concatenated single-copy core genes using the LG+F+I+G4 substitution model. Bee isolates form a monophyletic clade. The heat map indicates the genomic completeness of major metabolic pathways and functions related to energy and carbon metabolism, biosynthesis of amino acids, cofactors, and nucleosides, secretion, adhesion, and motility across the sequenced bacterial isolates. (B) Core genome phylogeny of the *Orbaceae* including the *Gilliamella* phylotype. The maximum-likelihood tree was computed on the concatenated amino acid sequences of 782 single-copy core genes using the JTTDCMut+F+I+G4 substitution model. The heat map indicates the genomic completeness of major metabolic pathways and functions related to energy and carbon metabolism, biosynthesis of amino acids, cofactors, and nucleosides, secretion, adhesion, and motility across the sequenced bacterial isolates. Download FIG S2, EPS file, 1.0 MB. Copyright © 2023 Sarton-Lohéac et al. 2023 Sarton-Lohéac et al. https://creativecommons.org/licenses/by/4.0/ This content is distributed under the terms of the Creative Commons Attribution 4.0 International license.

10.1128/mbio.03538-22.3 FIG S3 Core genome phylogeny of the *Acetobacteraceae*. The maximum-likelihood tree was computed on the concatenated amino acid sequences of 303 single-copy core genes using the LG+F+I+G4 substitution model. Bee isolates are distributed in two distant monophyletic clades. The heat map indicates the genomic completeness of major metabolic pathways and functions related to energy and carbon metabolism, biosynthesis of amino acids, cofactors, and nucleoside, secretion, adhesion, and motility across the sequenced bacterial isolates. Download FIG S3, EPS file, 0.9 MB. Copyright © 2023 Sarton-Lohéac et al. 2023 Sarton-Lohéac et al. https://creativecommons.org/licenses/by/4.0/ This content is distributed under the terms of the Creative Commons Attribution 4.0 International license.

Of the 46 strains selected for sequencing, 13 were not directly related to bacteria previously isolated from social bees. Most of these isolates matched minor ASVs in our community profiling analysis, with the exception of three strains (ESL0689, ESL0687, and ESL0729) (Fig. 2A). ESL0689 corresponded to the *Enterobacteriaceae* ASV19, which dominated the communities of all three Fv samples (20 to 35% of the reads per sample). This isolate was situated on a long branch diverging between the genera Klebsiella and *Raoultella* (Fig. S4). ESL0687 and ESL0729 corresponded to ASVs of the family *Streptococcaceae* which were detected in several stingless bee species in our studies as well as in previous studies (20, 37, 38). They formed a deep-branching sister clade of the flower-associated genus *Floricoccus* (Fig. S5A). All three strains seem to be novel species based on their divergence from previously sequenced bacteria and may represent specialized gut symbionts of stingless bees.

10.1128/mbio.03538-22.4 FIG S4 Core genome phylogeny of the *Enterobacteriaceae*. The maximum-likelihood tree was computed on the concatenated amino acid sequences of 303 single-copy core genes using the LG+F+I+G4 substitution model. In the metabolic heat map of the *Enterobacteriaceae* isolates, the genomes are ordered according to phylogeny. The heat map indicates the genomic completeness of major metabolic pathways and functions related to energy and carbon metabolism, biosynthesis of amino acids, cofactors, and nucleosides, secretion, adhesion, and motility across the sequenced bacterial isolates. Download FIG S4, EPS file, 0.8 MB. Copyright © 2023 Sarton-Lohéac et al. 2023 Sarton-Lohéac et al. https://creativecommons.org/licenses/by/4.0/ This content is distributed under the terms of the Creative Commons Attribution 4.0 International license.

10.1128/mbio.03538-22.5 FIG S5 (A) Core genome phylogeny of the *Streptococcaceae*, including *Floricoccus* isolates. The maximum-likelihood tree was computed on the concatenated amino acid sequences of 303 single-copy core genes using the LG+F+I+G4 substitution model. In the metabolic heat map of the *Streptococcaceae* isolates, the genomes are ordered according to phylogeny. The heat map indicates the genomic completeness of major metabolic pathways and functions related to energy and carbon metabolism, biosynthesis of amino acids, cofactors, and nucleosides, secretion, adhesion, and motility across the sequenced bacterial isolates. (B) Core genome phylogeny of the *Leuconostocaceae*. The maximum-likelihood tree was computed on the concatenated amino acid sequences of 303 single-copy core genes using the LG+F+I+G4 substitution model. Bee isolates are distributed in two distant monophyletic clades. In the metabolic heat map of the *Leuconostocaceae* isolates, the genomes are ordered according to phylogeny. The heat map indicates the genomic completeness of major metabolic pathways and functions related to energy and carbon metabolism, biosynthesis of amino acids, cofactors, and nucleoside, secretion, adhesion, and motility across the sequenced bacterial isolates. (C) Core genome phylogeny of the *Moraxellaceae*. The maximum-likelihood tree was computed on the concatenated amino acid sequences of 303 single-copy core genes using the LG+F+I+G4 substitution model. In the metabolic heat map of the *Moraxellaceae* isolates, the genomes are ordered according to phylogeny. The heat map indicates the genomic completeness of major metabolic pathways and functions related to energy and carbon metabolism, biosynthesis of amino acids, cofactors, and nucleosides, secretion, adhesion, and motility across the sequenced bacterial isolates. Download FIG S5, EPS file, 0.9 MB. Copyright © 2023 Sarton-Lohéac et al. 2023 Sarton-Lohéac et al. https://creativecommons.org/licenses/by/4.0/ This content is distributed under the terms of the Creative Commons Attribution 4.0 International license.

### Stingless bee gut bacteria have diversified into distinct species and reveal a high extent of genomic diversity.

**FIG 4:**
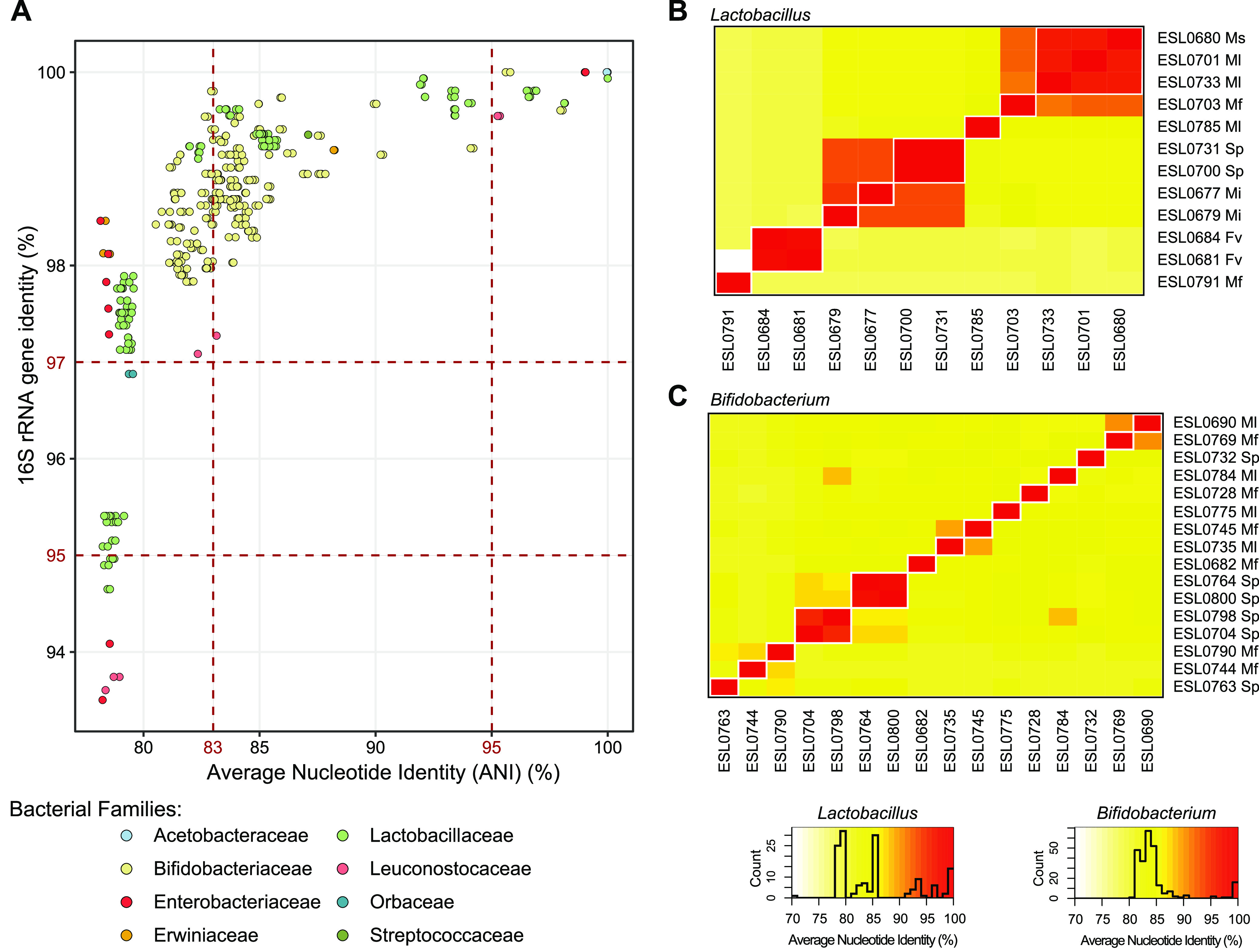
Genomic divergence among isolates with high 16S rRNA gene similarity. (A) The ANI versus 16S rRNA gene identity was plotted for each pair of isolate genomes belonging to the same bacterial family. The two vertical dashed bars indicate thresholds of 95% and 83%, demarcating the intraspecies and interspecies ANI values, respectively (86). The horizontal dashed lines delineate the commonly used species (>97%) and genus (>95%) thresholds for 16S rRNA gene similarity. (B and C) ANI heat maps of *Lactobacillus* Firm-5 isolates (B) and *Bifidobacterium* isolates (C). The 95% ANI clusters are outlined in white. The isolate host is indicated as follows: Fv, *Frieseomelitta varia*; Ms, *Melipona seminigra*; Ml, *Melipona lateralis*; Mf, *Melipona fuliginosa*; Mi, *Melipona interrupta*; Sp, *Scaptotrigona polysticta*.

Stingless bee isolates belonging to the same lineage were often separated by long branches in our phylogenies, indicating substantial genomic divergence (Fig. 3F and G; Fig. S2 and S6). This was confirmed by comparing pairwise 16S rRNA gene identity to genome-wide ANI between isolates of the same bacterial family. Despite high similarity in 16S rRNA gene identity, ANI was often <95%, suggesting that most lineages of stingless bee gut bacteria contain several divergent species (Fig. 4A). For example, the 12 sequenced strains of *Lactobacillus* Firm-5 fell into 8 distinct species-level clusters (i.e., ANI < 95%) (Fig. 4B). A similar pattern was observed for the 17 *Bifidobacterium* strains, which fell into 14 distinct species-level clusters (Fig. 4C), as well as the two *Gilliamella* and the two *Streptococcaceae* strains, which both also fell below the species-level ANI cutoff. Notably, some *Bifidobacterium* and *Lactobacillus* Firm-5 strains that were isolated from the same sample belonged to different ANI clusters, indicating that divergent bacterial species can co-occur in the same host species and colony. Inversely, strains belonging to the same ANI cluster were sometimes isolated from different bee species, suggesting that these bacterial species clusters are not necessarily host specific (Fig. 4B and C).

### Core microbiota members in stingless bees have functional capabilities similar to those of microbiota members in honey bees and bumblebees.

**FIG 5:**
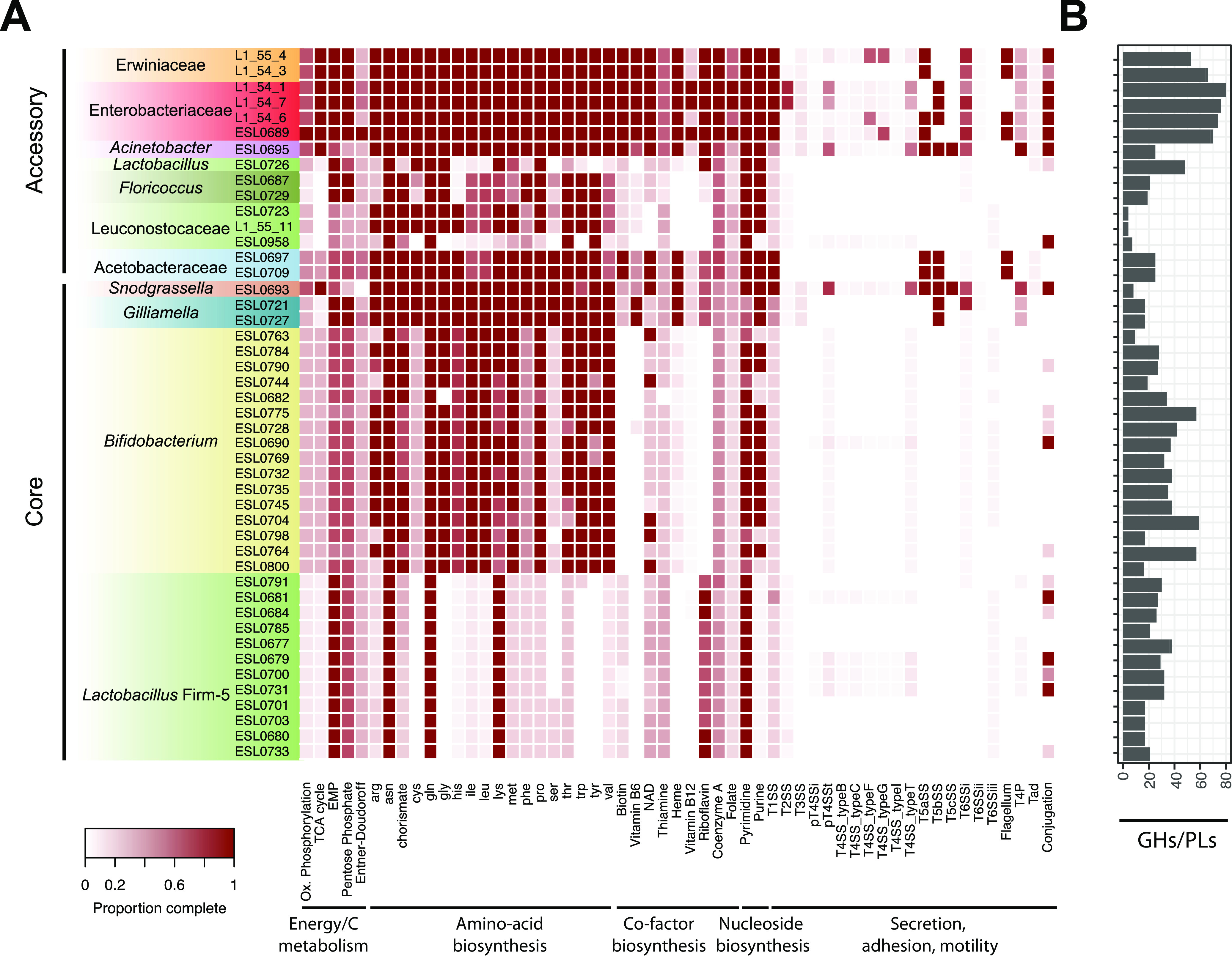
Metabolic capabilities of the sequenced stingless bee gut isolates. (A) The heat map indicates the genomic completeness of major metabolic pathways and functions related to energy and carbon metabolism, biosynthesis of amino acids, cofactors, and nucleosides, secretion, adhesion, and motility across the sequenced bacterial isolates. The isolates are grouped into core and accessory members based on whether they are related to bacteria isolated from other social bees. (B) Total numbers of glycoside hydrolase (GH) and polysaccharide lyase (PL) genes in each isolate.

To assess the functional potential of stingless bee gut bacteria, we determined the genomic completeness of different metabolic pathways and functions in the genomes of the sequenced strains and compared it to that of related bacteria which had been isolated from honey bees, bumblebees, or elsewhere and which were included in our phylogenomic analysis. We specifically looked at energy and carbon metabolism, amino acid, cofactor, and nucleoside biosynthesis, secretion, motility, and adhesion (Fig. 5).

### (i) Energy and carbon metabolism.

**FIG 6:**
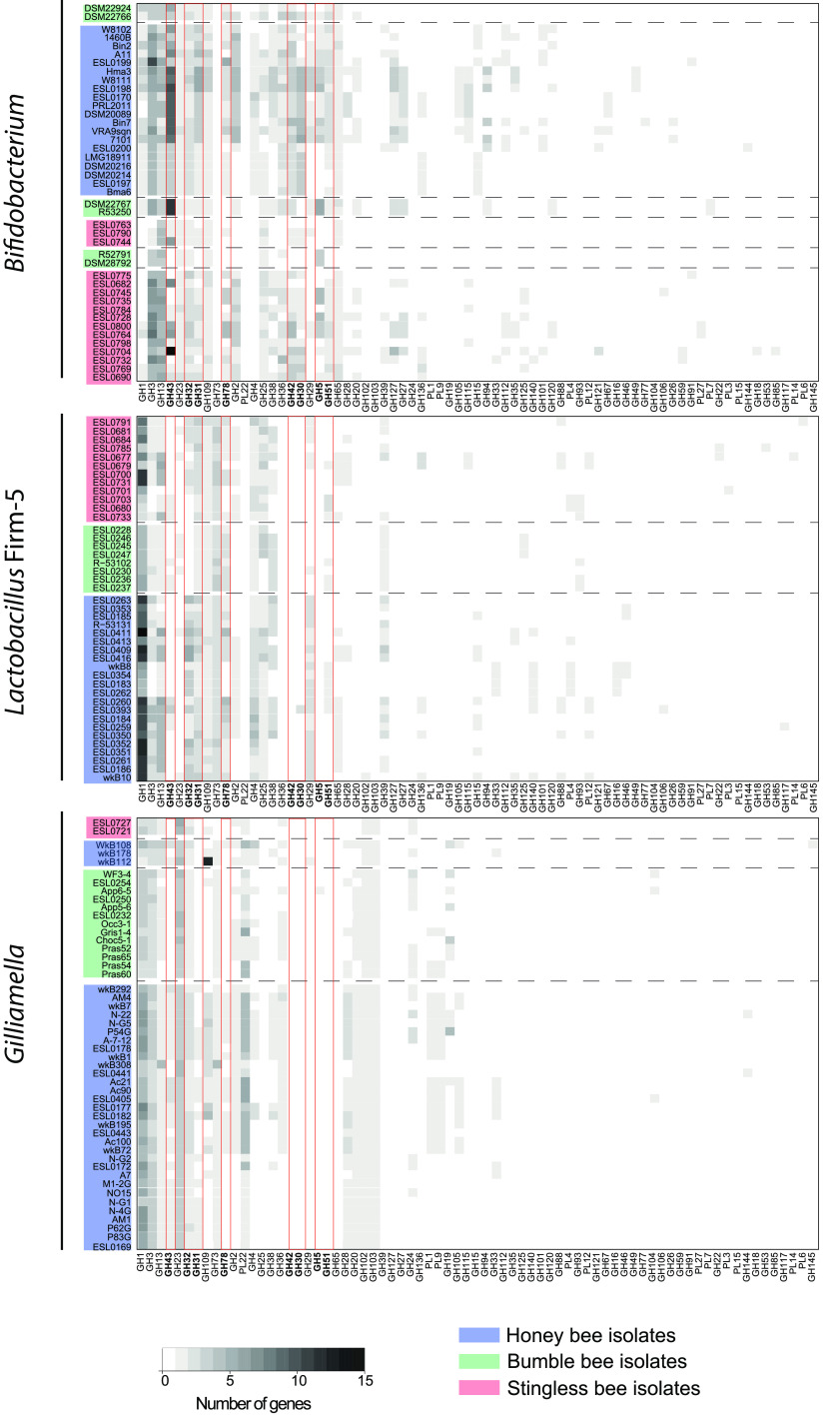
Carbohydrate-active enzyme profiles for *Bifidobacterium*, *Lactobacillus* Firm-5, and *Gilliamella* isolates. Distributions of genes in the GH and PL families for *Bifidobacterium*, *Lactobacillus* Firm-5, and *Gilliamella* isolates. The isolates were sorted according to their position in the phylogenies; the hosts are indicated by the background colors. Glycoside hydrolase families mentioned in the text are outlined in red. The color scale indicates the number of genes in each family.

Many of the sequenced strains (39/46), including all *Lactobacillaceae*, the *Bifidobacteriaceae*, and the two *Streptococcaceae* and *Gilliamella* strains, were missing key genes of the tricarboxylic acid (TCA) cycle and for oxidative phosphorylation but encoded functions for the breakdown (mostly glycoside hydrolases [GH]) and oxidation (Embden-Meyerhof-Parnas [EMP], pentose phosphate [PPP], and/or Entner-Doudoroff [ED] pathways) of sugars. A detailed analyses of the enzymes used for carbohydrate breakdown by the core members *Lactobacillus* Firm-5, *Bifidobacterium*, and *Gilliamella* showed that the stingless bee gut bacteria carried glycoside hydrolase family genes similar to those in honey bee and bumblebee isolates (Fig. 6), including enzyme families for cleaving plant-derived glycans. For example, the glycoside families GH5, GH30, GH31, GH42, GH43, and GH51 are involved in the degradation of hemicellulose (27, 30, 34). GH78 can be responsible for the cleavage of rhamnose residues from rutin, a major pollen-derived flavonoid that was demonstrated to be deglycosylated by honey bee isolates of *Lactobacillus* Firm-5 which carry GH78 genes (34). Another example is GH13, which includes neopullulanases and α-amylases for the breakdown of plant-derived starch. Together, these results suggest that stingless bee gut isolates of *Lactobacillus* Firm-5, *Bifidobacterium*, and *Gilliamella* are saccharolytic fermenters that break down pollen- or nectar-derived glycans, as previously reported for the corresponding bacteria in the gut of honey bees and bumblebees. Notably, there was substantial variation in the number and type of glycoside hydrolase family genes between divergent strains, which is in line with the extensive genomic diversity detected between stingless bee gut isolates of these three phylotypes. A complete TCA cycle was found only in the genomes of *Neisseriaceae* strain ESL0693, Acinetobacter strain ESL0695, the *Enterobacteriaceae*, and the *Erwiniaceae*. The same strains also harbored the most complete gene sets for oxidative phosphorylation. Notably, *Neisseriaceae* strain ESL0693 also lacked key genes in the EMP, PPP, and ED pathways and contained very few GH family genes (Fig. 5). This suggests that this bacterium cannot utilize sugars and obtains energy via aerobic respiration, as previously found for *Snodgrassella* isolates of honey bees and bumblebees (23).

### (ii) Amino acid, nucleoside, and cofactor biosynthesis.

Differences between stingless bee gut isolates of different taxonomic groups were also found in terms of their biosynthetic potential. Strains belonging to the *Lactobacillus* Firm-5 clade were auxotrophic for the production of most amino acids (i.e., all except for Lys, Gln, and Asn) as well as purine and several cofactors (e.g., heme and vitamins B_6_ and B_12_) (Fig. 5). Isolates of the *Bifidobacteriaceae*, *Streptococcaceae*, and *Leuconostocaceae* were also auxotrophic for many cofactors but for many fewer amino acids than *Lactobacillus* Firm-5. Interestingly, there was variation in auxotrophies among the bifidobacterial strains, especially for the production of purine, NAD^+^, Thr, Lys, Arg, Gly, and chorismate. Other strains (such as those of *Gilliamella*, *Snodgrassella*, *Acetobacteraceae*, *Enterobacteriaceae*, and *Erwiniaceae*) had fewer auxotrophies. Similar biosynthetic capability profiles were found in related strains included in our phylogenies, which suggests that these functional profiles are not specific to stingless bee gut bacteria but rather conserved across the entire phylotype (Fig. S2 to S6).

### (iii) Secretion, adhesion, and motility.

Secretion systems, pili, and flagella were mostly restricted to the Gram-negative bacteria of the isolated strains. Type I, type V, and type VI secretion systems were prevalent across these bacteria, whereas type II and type IV secretion systems were present in only a few strains (Fig. 5). Flagella were detected in the two *Acetobacteraceae*, all *Erwiniaceae*, and the *Enterobacteriaceae* strain ESL0689. Tad pili were not detected in any of the bacteria analyzed, while type IV pilus components were mostly found in the *Neisseriaceae* strain ESL0693 and Acinetobacter ESL0695 and to some extent also in *Orbaceae*, *Erwiniaceae*, and the *Enterobacteriaceae* strain ESL0689. Similar gene sets for secretion, adhesion, and motility were also found in related gut bacteria from honey bees or bumblebees, as shown by the functional profiles of all strains included in our phylogenies (Fig. S2 to S6).

Altogether, this first assessment of the gene content of the stingless bee gut bacteria shows that they have functional potential similar to that of isolates from honey bees and bumblebees, suggesting that they occupy similar ecological niches in the gut across social bees.

## DISCUSSION

Previous findings suggested that the core members of the bee gut microbiota were acquired in a common ancestor of the social bees (20) and possibly codiversified with their hosts over millions of years (16, 33). However, the lack of genomic data from gut bacteria of stingless bees has limited our view of the evolution of these specialized microbial communities. With the establishment of the genomes of diverse bacterial isolates from the stingless bee gut, our study fills an important knowledge gap and provides new lines of evidence that rule out strict codiversification between the core microbiota members and social bees.

Our genome-wide phylogenies of *Snodgrassella*, *Lactobacillus* Firm-5, and *Gilliamella* revealed that the identified lineages of stingless bee gut bacteria branched off before the divergence of the lineages of honey bee and bumblebee gut bacteria. This pattern is not congruent with the topology of the host phylogeny, in which honey bees diverged before the split of stingless bees and bumblebees (52, 53). The basal split of the stingless bee gut bacterial lineages depends on the rooting of our trees being correct. However, all phylogenies were supported by high bootstrap values at the critical nodes, suggesting robust phylogenetic signals in our data sets. Our phylogenies also showed that for both *Gilliamella* and *Snodgrassella*, honey bee isolates were not monophyletic, i.e., some lineages branched before and others after the divergence of the bumblebee clades, which is inconsistent with codiversification. This was already noted in a previous study (33), and similar results were also obtained for *Lactobacillus* Firm-5 based on the phylogenetic analysis of single protein-coding genes cloned from different social bee species (20). Finally, our phylogenomic analysis showed that honey bee and stingless bee isolates of *Bifidobacterium* belonged to two separate clades of the *Bifidobacteriaceae*, suggesting that these bacteria have independently adapted to the gut environment of social bees.

Codiversification can occur only when symbionts and hosts exhibit a high degree of partner fidelity and are transmitted vertically from one generation to the next over many generations (54). While most core microbiota members of social bees indeed seem to have a host-restricted distribution (20), examples of lineages with a broader host range exist as well (55). Moreover, some strains have been experimentally shown to be able to colonize nonnative hosts, demonstrating that host jumps are possible (20, 27). Our study showed that closely related stingless bee species (i.e., from the same or related genera) can have overlapping community profiles, with predominant ASVs being shared across hosts. Similar observations have been made in other 16S rRNA gene profiling studies of stingless bees (20, 37). While such analyses often provide insufficient resolution to discriminate between closely related strains or species, our genomic analyses confirmed that stingless bee isolates of *Bifidobacterium* and *Lactobacillus* do not necessarily cluster by host species. It is possible that the strength of host specificity varies across social bees or bacterial lineages, depending on the symbiotic function of the gut bacteria or the hosts’ divergence, ecology, or geographic distribution. This would influence the extent to which gut bacteria can codiversify within certain host lineages. To test this hypothesis, future studies would need to compare the strength of host specificity across multiple bees using representative sets of host species in each of the three main bee clades.

Another piece of evidence indicating that the microbiotas across social bees may be more variable than previously assumed comes from the observation that some of the designated core members were not always detected in the sampled bees. For example, while *Lactobacillus* and *Bifidobacterium* were prevalent across all six stingless bee species sampled in our study, they were absent from the gut microbial communities of some of the previously sampled bee species (15, 17, 37, 56). Likewise, stingless bees of the genus *Melipona* were shown to systematically lack the two core members *Snodgrassella* and *Gilliamella* (37). Both taxa were also rare across the four *Melipona* species analyzed in our study. However, three of 12 colonies analyzed had high abundances of *Snodgrassella*. This suggests that this bacterium is not completely absent from this bee genus but may occasionally be acquired from other bee species, that it varies in prevalence depending on season, bee age, or development, or that it is restricted to only the *Melipona* species analyzed in our study.

Finally, representatives of core members of the social bee gut microbiota were recently also found in bees of the distant genus *Xylocopa* (carpenter bees) (57, 58), suggesting that these bacteria may have been associated with bees before the emergence of sociality or that they have a broader and less specific distribution across bees than previously suspected.

In summary, our results together with previous findings indicate a rather dynamic evolutionary background of the core members of the social bee gut microbiota. Rather than their having strictly codiversified with their hosts, extended periods of host-restricted evolution (and likely codiversification in some lineages) seem to have been interrupted by host switches and by independent symbiont gains and losses. Our observation that the stingless bee isolates repeatedly form a sister group to bumblebee and honey bee isolates (for *Gilliamella*, *Snodgrassella*, and *Lactobacillus* Firm-5) is intriguing given the host phylogeny. It may suggest that these core members have an origin in stingless bees and then spread to the other two groups, especially for *Lactobacillus*, where two stingless bee isolates clades split before the split between bumblebee and honey bee isolates. However, given the large diversity of social and solitary bees, it is clear that the currently available data sets are insufficient to explain the distribution and phylogenetic relationships of these gut symbionts across hosts. Broader samplings of stingless bees, honey bees, and bumblebees, combined with genome-resolved approaches, are needed to fully understand the diversity, distribution, and evolutionary trajectories of social bee gut bacteria and to accurately reconstruct the ancestral bee microbiome composition. Formal analysis should be applied to test for codiversification.

The importance of sampling biases when assessing patterns of co-diversification between hosts and their gut bacteria is highlighted by the analyses of Bacteroidaceae gut symbionts of hominids. While originally reported to have codiversified with their hosts (59), reexamination with increased sampling disrupted the codiversification pattern observed earlier (60). In contrast, a recent study identified strong signals of parallel evolutionary history between seven (of 56 tested) gut bacterial taxa and human populations (61), and phylogenetic congruency has also been found for certain stinkbug insects and their primary gut symbionts (62). This demonstrates that codiversification has occurred between certain gut bacteria and their hosts.

Besides offering new insights into the evolution of the social bee gut microbiota, our genomic analysis also revealed the functional potential of major gut symbionts of the analyzed stingless bee species. All isolates of *Lactobacillus* Firm-5, *Bifidobacterium*, and *Gilliamella* carried genes for the saccharolytic fermentation of diet-derived carbohydrates. In contrast, *Snodgrassella* ESL0689 lacked such functions in its genome but instead harbored genes for aerobic respiration. These results are consistent with findings from honey bees and bumblebees (22, 23, 26–28, 30) and hence suggest that the core microbiota members occupy similar ecological niches across the three groups of social bees.

Another parallel to findings from honey bees and bumblebees was the extensive genomic divergence present among strains of the core members *Lactobacillus* Firm-5, *Gilliamella*, and *Bifidobacterium*, even when isolated from the same host species. Moreover, we found genomic variation in carbohydrate breakdown and amino acid and nucleoside biosynthesis functions among these strains. This suggests that the diversification of these bacteria has been driven not only by isolation in different host species but also by adaptation to different ecological niches in the gut, similar to what has been shown for bumblebees and honey bees (28, 33, 35, 63). These parallels may not be surprising, as the dietary preferences of the analyzed stingless bee species are similar to those of honey bees and bumblebees. It will be interesting to look at the functional potential of the core microbiota members in bees that have different dietary habits, such as the vulture bees (38) (i.e., stingless bees that feed on raw meat instead of pollen), yet share a subset of the core members with other social bees.

Some of the isolate genomes we sequenced in our study did not come from any of the core members of the social bee gut microbiota. They may represent transient community members, opportunistic pathogens, or host-specific gut symbionts with functions complementary to those of the core microbiota. Therefore, the established genomes present an important resource for future research. A particular strain that drew our attention was the *Enterobacteriaceae* strain ESL0689, as it belonged to an ASV that was present at high relative abundance in all three colonies of Fv. ESL0689 harbored a complete TCA cycle and a respiratory chain and could also synthesize most amino acids and cofactors, indicating a metabolic niche similar to that of *Snodgrassella* (23).

In conclusion, our study provides new insights into the evolution of the social bee gut microbiota and represents a first step toward characterizing the functional potential of major gut bacteria present in stingless bees. However, given the large diversity of stingless bees, with hundreds of different species distributed throughout the tropical and subtropical regions of the world, it is clear that our study presents only the starting point in characterizing their genomic diversity and functional potential. More detailed studies and larger genomic surveys combined with experimental analyses will be needed to understand their evolution and assess their impact on the host.

## MATERIALS AND METHODS

### Bee sampling.

Bees were collected from three different nests of each of the following Meliponini species in February and March 2019: *Frieseomelitta varia*, *Scaptotrigona polysticta*, *Melipona fuliginosa*, *Melipona interrupta*, *Melipona lateralis*, and *Melipona seminigra*. All nests were located in a rural meliponary in the vicinities of Iranduba municipality (Iranduba, AM, Brazil; 3°10′52.7′′S 60°07′08.5′′W), and the sampling was carried out under SISGEN collection permission no. A256E82. Bees were sampled at the entrance of each nest and immediately immobilized by cooling at 4°C. Then the entire gastrointestinal tract was dissected, and the hindgut separated from the anterior gut parts. Hindguts of bees from the same nest were pooled in two separate tubes. One pool per nest was mixed with 1× phosphate-buffered saline (PBS) and glycerol, homogenized using bead beating, and subsequently cryopreserved at −80°C. This pool was used for bacterial culturing as described below. The other pool was cryopreserved at −80°C without homogenization and was subsequently used for DNA extraction and 16S rRNA gene analysis. For the sample used for sequencing, 20 bees per nest were pooled, while for the sample for culturing, only 3 bees per nest were pooled. For *Frieseomelitta varia* and *Scaptotrigona polysticta*, we pooled 40 and 10 bees, respectively, due to the small size of these bee species.

### Bacterial culturing.

For establishing a culture collection of primary isolates from the gut of the six sampled bee species, serial dilutions of the cryopreserved homogenates were plated on eight different media: CBA (Columbia blood agar supplemented with 5 % defibrinated sheep blood [Thermo Fisher]), MRSA (De Man, Rogosa and Sharpe agar) supplemented with fructose (2%) and l-cysteine (0.1%), MRSA supplemented with mannitol (2%), chocolate agar, TSA (tryptone soy agar), TYG (tryptone glucose yeast extract agar), GC, LBA (Luria-Bertani agar) without NaCl, BHIA (brain heart infusion agar), and SDA (Sabouraud dextrose agar). Plates were incubated in two different conditions: in a microaerobic 5% CO_2_-enriched atmosphere and in an anaerobic chamber (72% N_2_, 8% H_2_, 20% CO_2_), both at 34°C. After 2 to 7 days of incubation, colonies of different size and appearance were picked and regrown on the same media with the same culturing conditions. Cryo-stocks of bacterial strains of interest were prepared by harvesting bacterial biomass in liquid media corresponding to the solid growth media and supplemented with 20% glycerol. For DNA isolation, bacteria were grown from the stocks, and a single colony was picked and regrown on fresh media before harvesting bacterial biomass.

### Genotyping of bacterial isolates.

All colonies that were selected for culturing were genotyped by PCR and Sanger sequencing of a 16S rRNA gene fragment. To this end, a small amount of bacterial material was transferred to a lysis buffer (1 M Tris-HCl [pH 7.5], 0.5 M EDTA, 10% SDS) containing 2.5 μL lysozyme (20 mg/mL) and 2.5 μL proteinase K (20 mg/mL) and incubated for 10 min at 37°C, 20 min at 55°C, and 10 min at 95°C. PCR was performed with universal bacterial primers that amplify the V1-V5 region of the 16S rRNA gene (27F [AGRGTTYGATYMTGGCTCAG] and 907R [CCGTCAATTCMTTTRAGTTT]) using the following reagents and thermocycler program: initial denaturing at 94°C for 5 min, followed by 32 cycles of denaturing at 94°C for 30 s, annealing at 56°C for 30 s, and extension 72°C for 1 min, and a final extension at 72°C for 7 min. PCR results were checked on a 1% agarose gel. PCR products selected for Sanger sequencing were purified using ExoSAP-IT (1 μL 5× ExoSAP, 4 μL double-distilled water [ddH_2_O]) with the following thermocycler program: 30 min at 37°C followed by 15 min at 80°C. Purified samples were then sent to Eurofins for sequencing. Sanger sequences were analyzed with Geneious suite (Geneious) and compared to GenBank at NCBI using BLAST tools (64).

### DNA isolation and genome sequencing.

DNA isolation for Illumina sequencing was carried out using a customized SPRI bead-based extraction method or the FastPure bacterial DNA isolation minikit (Vazyme). For the SPRI bead method, bacteria were harvested and resuspended in tubes containing 200 mg of 0.1-mm acid-washed glass beads and 200 μL of TER buffer (10 mM Tris-HCl, 1 mM EDTA, 100 μg/mL RNase A [pH 8.0]). Samples were homogenized using a FastPrep-25 5G instrument (2 rounds of 30 s with the power set to 6) and subsequently centrifuged at maximum speed for 10 min at room temperature. Forty microliters of SPRI beads was added to 100 μL of supernatant, immediately mixed thoroughly by repeated pipetting (>20 times), and incubated for 5 min at room temperature. After the tubes were placed on a magnet stand, the liquid was removed and discarded and the beads were washed twice with 200 μL 80% ethanol. After air drying of the tubes on the magnetic stand, 22 μL of 5 mM Tris-HCl (pH 8) was added. For isolating bacteria with the FastPure bacterial DNA isolation minikit, the manufacturer’s protocol for Gram-positive bacteria was followed.

DNA isolation for Oxford Nanopore Technologies (ONT) sequencing was carried out using a custom DNA extraction protocol for Gram-positive bacteria. Tubes were prepared with glass beads and 160 μL of buffer P1 (Qiagen). Then bacteria were harvested and resuspended in these tubes by intensive vortexing. Lysozyme (20 μL, 100 mg/mL) was added, and after gentle mixing, tubes were incubated at 56°C with shaking at 600 rpm for 30 min. Then, 4 μL RNase A (100 mg/mL) was added to the tubes, followed by 150 μL of buffer AL (lysis buffer; Qiagen). After mixing by vortexing, tubes were incubated in a thermal mixer (37°C, 900 rpm) for 20 min. Tubes were centrifuged for 10 min at 14,000 rpm to pellet the beads, and the supernatant was transferred to new tubes with 35 μL sodium acetate and 270 μL isopropanol and mixed by inverting. Following incubation for 1 h at 4°C, DNA was pelleted by centrifugation at 14,000 rpm for 10 min at 25°C. The supernatant was discarded, and the pellets were washed with 1 mL 80% EtOH. After a second centrifugation (14,000 rpm for 10 min at 25°C), the ethanol was removed, and the pellet was left to dry at room temperature. DNA pellets were solubilized with 50 μL TER (10 mM Tris-HCl, 1 M EDTA [pH 8.0], 2 mg/mL RNase A), and tubes incubated at 37°C for 15 min. The solution was then transferred to PCR tubes. Forty microliters of NGClean beads was added, and the solution was mixed by repeated pipetting. After a 5-min incubation, PCR tubes were placed on magnetic stands. When the solutions were clear, the liquid was removed, and the beads were washed twice with 200 μL of 80% EtOH. Upon complete drying, beads were resuspended with 22 μL of 5 mM Tris-HCl (pH 8.0). Finally, tubes were placed again on the magnetic stand, and when the solution were clear, the supernatant was transferred to new 1.5-mL Eppendorf tubes.

Illumina sequencing libraries were prepared using the Nextera DNA Flex library preparation kit following instructions of the Illumina reference guide. This was followed by indexing, dilution, and denaturation according to the Illumina documents Index Adapters Pooling Guide and the MiniSeq System Denature and Dilute Libraries Guide. Libraries were checked and quantified using a double-stranded DNA (dsDNA) fluorescent dye, before being loaded on a MiniSeq high-output flow cell (150PE). ONT libraries were prepared using the ligation-based approach (LSK109). The sequencing was conducted on a ONT MinION instrument for a duration of 72 h with high-accuracy base-calling using Guppy (v5.0.11).

### Genome assembly.

Forty-six isolates from the stingless bees were sequenced with Illumina MiniSeq (150PE). Raw reads were checked using FastQC v0.11.9 (65) and trimmed by Trimmomatic v0.39 (66) using the parameters: PE -phred33 AllIllumina-PEadapters.fa:3:25:7 LEADING:9 TRAILING:9 SLIDINGWINDOW:4:15 MINLEN:60. For *de novo* assembly, we used SPAdes (–careful option, v3.15.2) (67). For assembly quality control, reads were mapped back against the assembly with BWA v0.7.17 (68) and SAMtools v1.12 (69) and plotted with R v4.1.1. Genomes completeness was evaluated with checkM v1.0.13 (70).

Additionally, 26 isolates were also sequenced with Oxford Nanopore to produce long reads. Nanopore long reads were filtered with Filtlong v0.2.0 (https://github.com/rrwick/Filtlong) for a minimum length of 7,000 and minimum mean *q* score of 10 (min_length 7000, min_mean_q 10, length_weight 10). ONT-based assemblies were computed with Flye v2.7.1 (71) over 5 iterations. Graphmap v0.5.2 (72) and Racon v1.0.1 (73) were used to perform two rounds of polishing. Finally, the Racon-corrected assembly and Illumina reads were fed to Pilon v1.24 (74) for single-base and indel corrections.

### Genome annotation and analysis.

Genomes were annotated with Prokka v1.13 (75). Phylogenies were computed for the bacterial families for which we had an isolate. For each family, we identified a set of closely related strains and outgroup taxa and retrieved their genomes from NCBI and IMG/Mer (76). All genomes were reannotated with Prokka to ensure annotations consistency. Gene orthology was inferred with OrthoFinder v2.3.8 (77). Single-copy ortholog genes were selected, and their amino acid sequences were aligned (mafft v7.453) (78). An in-house script was used to trim the alignments by removing positions with more than 50% gaps, and sequences belonging to the same genome were concatenated to produce a core gene alignment. This alignment was used to infer the maximum-likelihood phylogeny using IQTree (v1.7.beta17, -st AA -bb 1000 -seed 12345 -m TEST) (79). For each phylogeny, the best evolutionary model was chosen according to the Bayesian information criterion (BIC): LG+I+G4, *Enterobacteriaceae*; LG+F+I+G4, *Acetobacteraceae*, *Bifidobacterium*, *Lactobacillus*, *Leuconostocaceae*, *Moraxellaceae*, *Neisseriaceae*, and *Streptococcaceae*; and JTTDMut+F+I+G4, *Orbaceae*. Branch support of the trees was inferred using 1,000 ultrafast bootstrap (UFBoot) repetitions. Clades can be trusted when UFBoot values are >95%.

### 16S rRNA gene based community profiling.

Region V4 of the 16S rRNA gene was amplified using the primers 515F-Nex and 806R-Nex (TCGTCGGCAGCGTCAGATGTGTATAAGAGACAGGTGCCAGCMGCCGCGGTAA and GTCTCGTGGGCTCGGAGATGTGTATAAGAGACAGGGACTACHVGGGTWTCTAAT). The primers include Nextera XT index adapter sequences and the primers for the 16S rRNA V4 region (80) described by Kešnerová et al. (22). The two-step PCR was performed as follows: the first PCR used 12.5 μL of 2× Phanta Max master mix (Vazyme, Nanjing, China), 5 μL of Milli-Q water, 2.5 μL of each primer (5 μM), and 2.5 μL of template DNA for a total volume of 25 μL. The PCR program started with a denaturation step at 98°C for 30 s, followed by 25 cycles of amplification (10 s at 98°C, 20 s at 55°C, and 20 s at 72°C) and a 5-min final extension step at 72°C. The PCR products were verified by 2% agarose gel electrophoresis, purified with clean next-generation sequencing (NGS) purification beads in a 1:0.8 ratio of PCR product to beads and eluted in 27 μL Tris (10 mM, pH 8.5). A second PCR step was performed to append the unique dual indexes to each sample in a total volume of 25 μL using 12.5 μL of 2× Phanta Max master mix (Vazyme, Nanjing, China), 5 μL of Milli-Q water, 2.5 μL of Nextera XT index primers 1 and 2 (Nextera XT Index kit, Illumina), and 2.5 μL of templated DNA. The PCR program started with a denaturation step at 95°C for 3 min, followed by 8 cycles of amplification (30 s at 95°C, 30 s at 55°C, and 30 s at 72°C), and a 5-min final extension step at 72°C. The libraries were then cleaned using clean NGS purification beads (1:1.1 ratio of PCR product to beads) and were eluted in 27.5 μL Tris (10 mM, pH 8.5). Prior to sequencing, the PCR product concentrations were quantified by PicoGreen and pooled in equimolar concentrations; the negative controls and blank extractions were pooled in equal volume. Sequencing was performed on an Illumina MiSeq sequencer (2 × 250 bp) by the Genomic Technology Facility of the University of Lausanne. We followed the DADA2 (81) pipeline to analyze the sequencing data. For the first part of the analysis, we executed the pipeline only on the 18 samples from our study. To control for possible contaminants, we used blank extractions and water as negative controls during the PCR. For the second part of the analysis, where we combined our data set with the one from reference 20, we executed the pipeline independently a second time. Sequence quality control was performed with the DADA2 integrated function plotQualityProfile. Data from the three sequencing runs were processed independently for the filtering, dereplication, the error rate calculation, and the sample inference. After merging the denoised forward and reverse pairs, we merged the three sequence tables (mergeSequenceTables), and we applied the collapseNoMismatch function to unite similar ASVs with shifts or length variation. We selected sequences in the range from 250 to 256 bp and removed chimeric sequences. The Silva nonredundant small-subunit (SSU) database v138.1 (81) was used for the taxonomic assignment of the ASVs. We filtered out 62 ASVs for which the taxonomic assignment matched “Eukaryota,” “Chloroplast,” or “Mitochondria.” Finally, we removed samples with fewer than 5,000 reads. NMDS plots were computed in R using Bray-Curtis distances (Phyloseq: ordinate). The adonis package was used to carry out PERMANOVA.

### Functional profiler.

We developed a genomic profiler using several software programs to annotate the genomes and compute the completeness of key pathways and functions. The genomes were reannotated with GhostKOALA (82) to obtain KEGG annotations of genes. We created a set of rules to define the steps of selected energy and metabolism pathways and cofactor/nucleoside biosynthesis pathways and computed their completeness. In brief, an in-house script parsed the KEGG annotations of the genomes and for each pathway evaluated the completeness of each step. The pathway completeness was then summarized by counting the number of steps present relative to total number of steps needed. To obtain the completeness of amino acid biosynthesis pathways, we used GapMind (83). Secretion systems and related appendages were detected in the genomes by MacSyFinder’s TXSScan module (84). Finally, we ran dbCan (85) for the annotation of carbohydrate-active enzymes.

### Data availability.

The bacterial genomes and sequencing data are available in the NCBI’s BioProject database under the accession number PRJNA906295. Scripts used for the amplicon sequencing analysis, the phylogenies, and the metabolic profiler can be found on GitHub https://github.com/gsartonl/Publication_Sarton-Loheac_2022.

10.1128/mbio.03538-22.6 FIG S6 (A) Core genome phylogeny of the genus *Lactobacillus*, including bacteria belonging to the social bee-specific phylotype *Lactobacillus* Firm-5, highlighted in different colors according to the host species/group. The maximum-likelihood tree was computed on the concatenated amino acid sequences of 355 single-copy core genes using the substitution model LG+F+I+G4. In the metabolic heat map of the *Lactobacillus* isolates, the genomes are ordered according to phylogeny. The heat map indicates the genomic completeness of major metabolic pathways and functions related to energy and carbon metabolism, biosynthesis of amino acids, cofactors, and nucleosides, secretion, adhesion, and motility across the sequenced bacterial isolates. (B) Core genome phylogeny of the genus *Bifidobacterium* with bacteria belonging to social bee-specific clades. The maximum-likelihood tree was computed on the concatenated amino acid sequences of 151 single-copy core genes using the substitution model LG+F+I+G4. In the metabolic heat map of the *Bifidobacterium* isolates, the genomes are ordered according to the phylogeny. The heat map indicates the genomic completeness of major metabolic pathways and functions related to energy and carbon metabolism, biosynthesis of amino acids, cofactors, and nucleosides, secretion, adhesion, and motility across the sequenced bacterial isolates. Download FIG S6, EPS file, 1.1 MB. Copyright © 2023 Sarton-Lohéac et al. 2023 Sarton-Lohéac et al. https://creativecommons.org/licenses/by/4.0/ This content is distributed under the terms of the Creative Commons Attribution 4.0 International license.
